# The Effects of the Factors Related to the Patient and the Disease on the Performance of Ablation Therapy in Patients with Differentiated Thyroid Cancer who have Received I-131 Ablation Therapy

**DOI:** 10.4274/Mirt.25744

**Published:** 2012-12-20

**Authors:** Tarık Şengöz, Erdem Sürücü, Yusuf Demir, Erkan Derebek

**Affiliations:** 1 Balıkesir Government Hospital, Department of Nuclear Medicine, Balıkesir, Turkey; 2 Yüzüncü Yıl University, School of Medicine, Department of Nuclear Medicine, Van, Turkey; 3 Izzet Baysal Government Hospital, Department of Nuclear Medicine, Bolu, Turkey; 4 Dokuz Eylül University, School of Medicine, Department of Nuclear Medicine, İzmir, Turkey

**Keywords:** Thyroid neoplasms, Iodine radioisotopes, prognosis

## Abstract

**Objective:** To investigate whether the factors related to the patient and the disease have any effect on the success of ablation therapy in patients with differentiated thyroid cancer who have received I-131 ablation therapy.

**Material and Methods:** All the patients with differentiated thyroid cancer were referred for I-131 ablation therapy after thyroidectomy between July 2007 and September 2009. The patients had at least six months of follow-up. Age, gender, type of tumor, presence of capsule invasion, size of tumor, number of the tumors, localization of the tumor, invasion of thyroid capsule, lymph/vessel invasion, presence of metastatic lymph nodes, type of surgery, preablation values of thyroglobulin (Tg), AntiTg, TSH, surveys for the evaluation of metastatic disease, (thyroid and bone scintigraphy, neck and abdominal ultrasonography, chest and brain computerized tomography), administered dose, postablation I-131 whole body scan (WBS) and diagnostic I-131 WBS, neck USG, values of Tg and AntiTg at the 6th month were recorded. The presence of residual thyroid activity on the 6th month diagnostic I-131 WBS image was accepted as the criterion for ablation success.

**Results:** 191 patients with differentiated thyroid cancer were assessed in this study. The overall success rate of the first ablation therapy was 74.3%. The success rate of the ablation therapy was 66% and 75% in metastatic group and non-metastatic group, respectively. Except the significant correlation between the number of pathologic lymph nodes and the success of ablation (p=0.025), there was no other significant correlation between the patient/disease related factors and the success of ablation therapy.

**Conclusion:** Significant correlation between the number of the pathologic lymph nodes and the ablation therapy performance can also be due to statistical error because of the limited sample size. There was no significant correlation between other patient/disease related prognostic factors and the success of ablation therapy.

**Conflict of interest:**None declared.

## INTRODUCTION

Thyroid cancer is the one of rare type of cancers in humans that constitutes less than 1% of all cancers (1). However, it has the highest mortality rate among the endocrine cancers (2). Differentiated thyroid cancer (DTC) is evaluated and treated according to the prognostic factors. Many prognostic scoring systems (TNM, AMES, AGES, MACIS, etc.) have been developed for the evaluation. The purpose of the scoring systems is to make the separation of low-and high-risk patients. Mortality and recurrence rate is very low in patients with low-risk. 10-year and 20-year mortality rate in the high risk group is 20-30% and 40% (3). Prognostic factors include age, gender, type of surgical treatment, tumor size, tumor type, being multifocal / multicentric, thyroid capsule invasion, lymph/blood vessel invasion, lymph node (LNs) and distant metastases.

The efficacy of I-131 ablation therapy after surgery is still controversial, but it is reported that ablation therapy reduces morbidity and mortality. Mazzaferri et al. reported that recurrence rate after ablation was 16% in the patients with a tumor size greater than 1.5 cm, multifocal, capsule or LNs invasion, on the other hand, the recurrence rate after TSH supression therapy was 38%; mortality rate was 3% and 8%, respectively in their study with 802 patients (4). The effect of the prognostic factors and patient characteristics on the success of ablation therapy was investigated in some studies, but there is still debate in the effect of prognostic factors and patient characteristics.

In this study, the effect of clinical and laboratory findings of the patient and the disease on the ablation success was investigated by the follow-up (6^th^ month, 1^st^ year, 2^nd^ year) of patients who received ablation therapy after surgical treatment of DTC.

## MATERIALS AND METHODS

**Patients**

Patients who were diagnosed with DTC, treated with total and/or near-total thyroidectomy and referred for I-131 ablation therapy between July 2007 and September 2009 were included in our study. Each patient was followed-up at least 6 months. Age, gender, tumor type, the presence of tumor capsule and tumor capsule invasion, tumor size, number of tumors, tumor localization, thyroid capsule and lymphatic / vascular invasion, the presence of LNs metastasis, type of surgery, pre-ablation Thyroglobulin (Tg), anti-thyroglobulin (ATG) and TSH values, imaging for metastatic screening (thyroid and bone scintigraphy, neck and abdominal ultrasonography, chest and brain CT), the administered dose, post-ablation I-131 whole body scan (WBS) and diagnostic I-131 WBS, neck ultrasonography, Tg, ATG results at 6th months were recorded.

**Therapy and Follow-up**

Standard dose was administered to all patients: 100 mCi to the patients with low-risk and without LNs or distant metastasis, 150-175 mCi to the patients with invasion to the thyroid capsule, lymph / blood vessels, surrounding soft tissue and without LNs or distant metastasis, 150-175 mCi to the patients with high risk and LNs metastasis at diagnosis, 200 mCi to the patients with high risk and distant metastases that was detected with imaging modalities (bone scan, brain and chest CT, neck and abdominal USG). 50 mCi dose was given to 2 patients due to the large amount of residual tissue and 75 mCi dose was given to 3 patients secondary to the diagnosis of microcarcinoma.

Patients were followed with thyroid function tests (TFT) for the regulation of suppression dose during the first 3 months. Ablation success was evaluated six months after therapy. During this period, Tg and ATG values one month after stopping T4 suppression therapy, neck ultrasonography and diagnostic WBS with 10 mCi were obtained. The ablation success was evaluated according to the diagnostic WBS in the 6th month. It was concluded that the patients without any residual uptake in the thyroid region in WBS were ablated successfully. Mild uptake in the thyroid region was evaluated as an unsuccessful ablation.

**Statistical Analysis**

Mann-Whitney U test was used for the effect of these variables to the success of ablation because of the non-homogeneous distribution. Chi-square test was used for the relationship between categorical variables and the success of ablation. In addition, logistic regression analysis was performed for the evaluation of the cumulative effects of these variables on the success of the ablation. In the univariate analysis method, two different models was created using the variables associated with the success of ablation and the variables identified in the literature associated with the success of ablation.

The presence of the metastatic LNs (absent/present) was used in one of the models and the number of the metastatic LNs (0-1, >2) was used in the other model. Furthermore, age, gender, tumor type, the number of tumor foci, thyroid capsule invasion, lymph / blood vessel invasion were added to the both models.

## RESULTS

Descriptive characteristics of 191 (150 Women, 41 Men; average age: 45±13 years) patients are given in Table 1. Ablation therapy was successful in 142 of 191 patients (74.3%) and unsuccessful in 49 of 191 patients (25.7%). Significant difference was seen in the success of ablation between the patients with two or more metastatic LNs and 0-1 metastatic LNs (p<0.05). Successful ablation was achieved in 138 patients (76.2%), with 0-1 metastatic LN and in four patients (40%) with two or more metastatic LNs.

The ablation success rate decreases with increasing number of metastatic LNs. Significant relationship was not found between the ablation success and the categorical variables that belong to the patient and disease (Table 2). Significant difference was not seen between the ablation success and the categorical variables that belong to the patient and disease. There was also no significant difference between the ablation success and the numeric variables (p>0.05). The average and standart deviations of the numeric variables in the groups with successfull and unsuccessfull ablation were shown in Table 3.

**The Results of Multivariate Analysis**

Significant difference was found only for the number of metastatic LNs among all variables. Additionally, it was revealed that the ablation success rate reduces by 80% in the patients with one or more metastatic LNs (Table 4).

**The Relationship Between the Success of the Ablationand Tg and ATG**

The average Tg value at the 6th month was found as 9.3±43.3 in the group with unsuccessful ablation and 1.9±14.4 in the group with successful ablation. There was a significant relationship between the ablation success and Tg value at the 6th month (p<0.05). The average ATG value at the 6th month was found as 21.5±6.8 in the group with unsuccessful ablation and 30.5±40.8 in the group with successful ablation. No significant relationship was found between the ablation success and ATG value in 6th month (Table 5). No significant relationship was seen between the degree of residual scale and some of categorical variables (p> 0.05) (Table 6).

**The Ablation Success in Patients with Metastatic andNon-metastatic Disease Before the Therapy**

There was no metastatic disease in 176 of 191 patients (%91). Significant difference was not detected between the ablation success and categorical/numerical variables in patients with non-metastatic disease. Logistic regression model as a multivariate analysis method has been developed in order to see the cumulative effect of these factors to the success of ablation and there was no significant difference in this model (Table 7). High-dose I-131 therapy (175 and 200 mCi) were given to ? of (15) 191 patients (7.8%) with lymph node/distant metastasis before ablation therapy. There was no significant diference between the ablation success and categorical/numerical variables in the patients given high-dose, except the number of metastatic lymph nodes in neck ultrasonography.

## DISCUSSION

The main purpose of the I-131 ablation therapy is removing the residual tissue in the patients with DTC (5,6). Microscopic tumor foci are eradicated as a result of ablation (6). The elimination of all residual tissue with ablation increases the sensitivity of WBS by increasing the accumulation of I-131 in the metastatic foci (7). Furthermore, ablation therapy also increases the sensitivity and specificity of Tg values that indicates the recurrent disease during follow-up (8). It has been reported that ablation therapy reduces recurrence, metastasis, and mortality rate dramatically (4,6,9). There are many factors that affect the ablation success. It has been reported that absorption and transport of radioiodine to the thyroid cells, NIS expression heterogeneity, the radiosensitivity of thyroid cell, TSH levels, the amount of residual tissue, the amount of the dose can affect the ablation success.

In this study, the ablation success was found as 74.3%. There are different results for the ablation success in the studies. The ablation success was reported as 70-95% in the literature. Zidan et al. found the ablation success as 94% by giving 30-85 mCi dose in 238 patients and they also reported that there was no significant difference between doses (10). In the study with 149 patients, ablation success was found as 72.8% with the doses of 30-155 mCi and they also indicated that the ablation success was better with doses higher than 50 mCi (11). In our study, 72.8% of the patients were given 100 mCi, 16.8% of the patients were given 150 mCi, 6.3% of the patients were given 175 mCi, and 1.5% of the patients were given 200 mCi. There was no significant difference between the amount of the dose and the ablation success. Most of the literature reported that there was no significant difference in the ablation success between low-dose and high-dose iodine (12,13,14,15). Therefore, this difference may be secondary to the heterogeneity of NIS expression in residual tissue and as a result of this, the difference of iodine uptake in these patients (16).

In the present study, no significant difference was found between the ablation success, gender and average age of the patients and the results of this study are consistent with the literature (17,18,19). In our study, ablation was successful in 74.7% of patients with papillary cancer and in 71.4% in patients with follicular cancer. There was no significant correlation between tumor type and the success of ablation.

In the present study, no significant difference was seen between the tumor size and the ablation success. While tumor diameter has significant effects on the recurrent disease, it has no effects on the success of ablation. Sirisalipoch et al. and JD Lin et al. showed that the tumor size did not affect the ablation success (17,19).

There was no significant correlation between the ablation success and the presence of the thyroid capsule invasion. There is no data showing that relationship between the thyroid capsule invasion and success of ablation in the literature. However, Mazzaferi et al. showed that thyroid capsule invasion affects the therapy response and increases the mortality in their study with 1355 patients (20).

There was inverse and effective relationship between the ablation success and the number of metastatic lymph nodes in our study. Increasing the number of metastatic LNs (from 0 to 1 or from 1 to 2) reduces the ablation success by 80%. In some studies, it has been reported that tumor metastasis to lymph nodes is an important prognostic risk factor in the recurrent disease and cancer-specific mortality. There are no studies including the explaination of this relationship in the literature. However, it has been known that NIS expression and iodine uptake are lower in the tumor foci and metastatic lymph nodes than the normal thyroid tissue (16). Kyoung So et al. showed NIS expression in the primary tumor focus (95%) and metastatic lymph nodes (96%). But, they reported that NIS glycoprotein was located in the cytoplasm and only 15% of this was expressed in the basal membrane which might result in unsuccess ablation (21). In our study, since the ablation of residual tissue is evaluated, we thought that the lower rate in the success of treatment might be due to the microscopic tumor foci in residual tissue in the patient with metastatic lymph nodes (16).

Because the number of patients with metastatic LNs are relatively smaller than the number of patients without metastatic LNs (20 patients positive), false statistical results might be considered. Therefore, further studies are needed to support our findings.

Significant correlation was found between the Tg value at the 6th month and the ablation success. The possibility of the ablation success decreases when Tg value increases. There are also a few studies supporting this finding in the literature (18,19).

In the current study, it was shown that the detection of LN metastasis reduces the ablation success. It has been reported that metastasis can cause poor therapy response and poor prognosis (22). Ablation dose accumulates in both metastatic and residual tissue in patients with metastatis and higher doses that were preferred in order to provide ablation can be insufficient for the ablation however, statistical errors can not be excluded due to the low number of patients with metastases.

## CONCLUSION

I-131 ablation therapy is a successful form of treatment that aims to destroy the remaining residual tissue after surgical treatment in the patients with differentiated thyroid cancer.

There is no significant correlation between the ablation success and age, gender, tumor type, presence and invasion of tumor capsule, thyroid capsule invasion, tumor size, number of tumor foci, the lymph / blood vessel invasion, metastatic lymph node and the presence of residual tissue in neck ultrasonography, the number of residual foci in thyroid scintigraphy, preablation TSH, Tg, and ATG values, the amount of the dose given to the patient. The success of ablation is independent from these variables.

Significant correlation exists between the success of ablation and the number of metasatic lymph nodes. However, this finding can also be considered to be a statistical error due to the small sample size of the patients.

## Figures and Tables

**Table 1:**
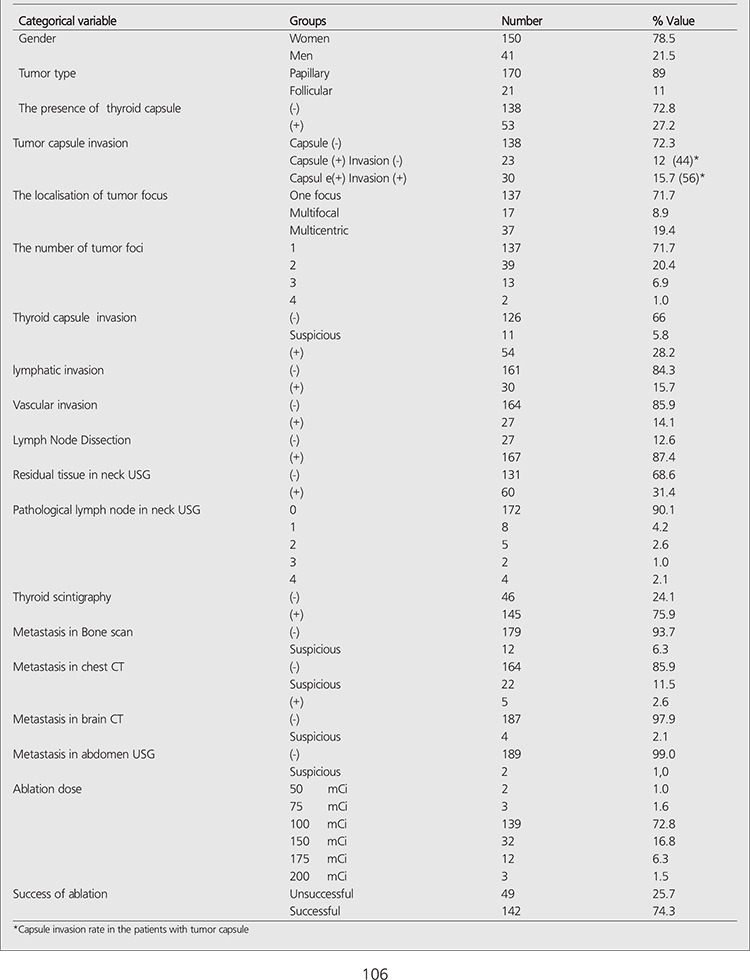
Descriptive characteristics and the Success of ablation

**Table 2:**
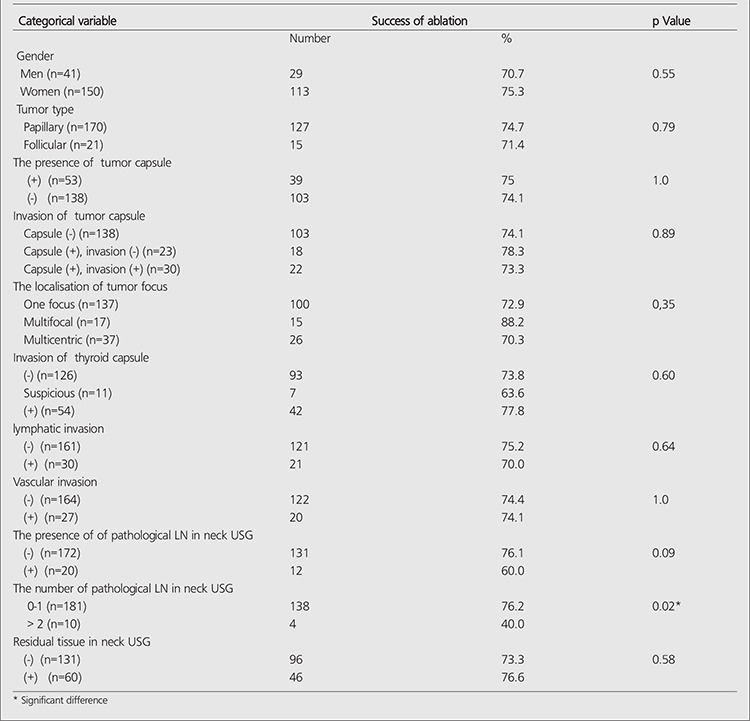
The relationship between the success of ablation and patients/disease releated factors (Categorical variable)

**Table 3:**
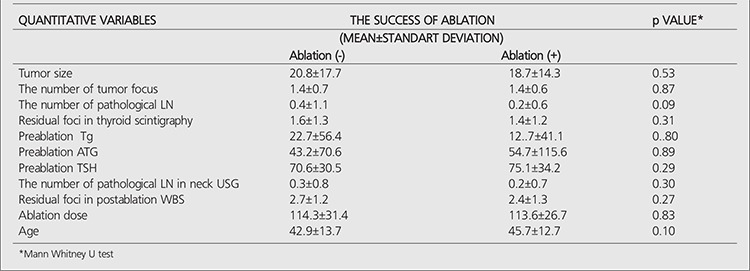
The relationship between the success of ablation and patients/disease releated factors (Quantative variables)

**Table 4:**
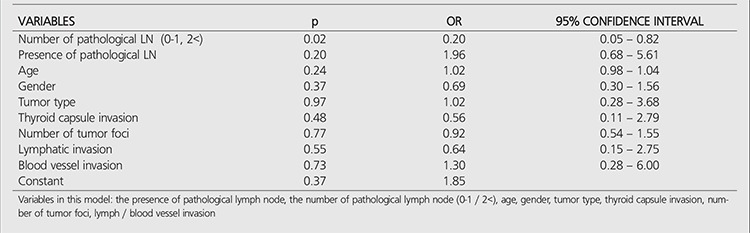
Multivariate analysis (logistic regression)

**Table 5:**
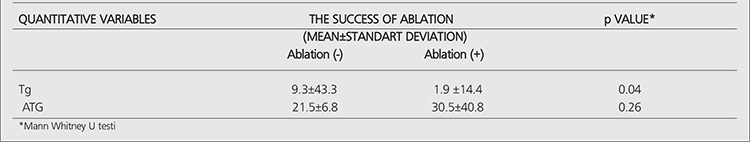
The relationship between the success of ablation and Tg/ATG values in 6^th^ month

**Table 6:**
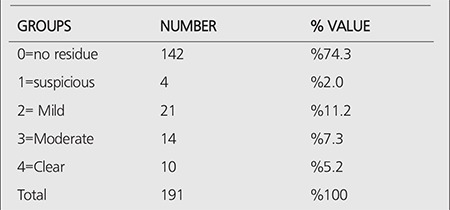
Descriptive findings of the degree of residual scale in the I-131WBS in 6^th^ month

**Table 7:**
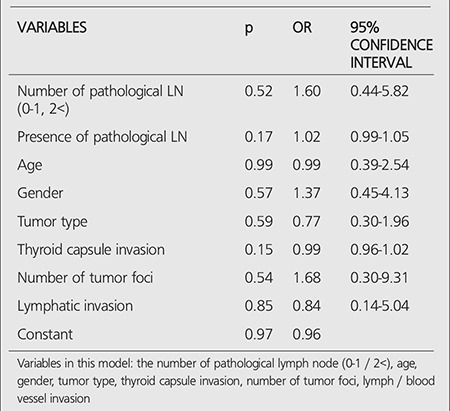
The results of multivariate analysis of metastasis in patients withnon-metastatic disease
